# The moderating role of social network size in the temporal association between formal social participation and mental health: a longitudinal analysis using two consecutive waves of the Survey of Health, Ageing and Retirement in Europe (SHARE)

**DOI:** 10.1007/s00127-020-01961-2

**Published:** 2020-10-09

**Authors:** Ziggi Ivan Santini, Paul E. Jose, Ai Koyanagi, Charlotte Meilstrup, Line Nielsen, Katrine R. Madsen, Carsten Hinrichsen, Robin I. M. Dunbar, Vibeke Koushede

**Affiliations:** 1 The Danish National Institute of Public Health, University of Southern Denmark, Studiestræde 6, 1455 Copenhagen, Denmark; 2 School of Psychology, Victoria University of Wellington, PO Box 600, Wellington, 6012 New Zealand; 3 Parc Sanitari Sant Joan de Déu, Universitat de Barcelona, Fundació Sant Joan de Déu, CIBERSAM, Dr Antoni Pujadas, 42, 08830 Sant Boi De Llobregat, Barcelona Spain; 4 ICREA, Pg. Lluis Companys 23, Barcelona, Spain; 5 Department of Experimental Psychology, University of Oxford, Radcliffe Observatory, Anna Watts Building, Woodstock Rd, Oxford, OX2 6GG UK; 6 Department of Psychology, University of Copenhagen, Øster Farimagsgade 2A, 1353 Copenhagen, Denmark

**Keywords:** Social participation, Social network, Quality of life, Depression, Aging

## Abstract

**Introduction:**

Previous studies have shown that engaging in formal social participation may protect against declining mental health, but social network size (the number of close social ties a person has) may moderate the relationship. We assessed the potential moderating role of social network size using longitudinal data.

**Methods:**

Nationally representative data from two consecutive waves (2011, 2013) of the SHARE survey were analyzed. The data consisted of 38,300 adults from 13 European countries aged 50 years and older in 2011. Measures pertaining to formal social participation, social network size, quality of life, and depression symptoms were used. Multivariable linear regression models were conducted.

**Results:**

The majority of participants (over 70% of the sample) had a social network size of four or less close social ties. We identified significant moderations in both models. Individuals with relatively few close social ties may have benefitted from formal social participation both in terms of reductions in depression symptoms and increases in quality of life, while formal social participation among those with many social ties did not appear to be beneficial, and may even to some extent have been detrimental.

**Conclusions:**

Declines in mental health specifically among those with relatively few close social ties could potentially be prevented through the promotion of formal social participation. It is possible that such strategies could have a greater impact by specifically targeting individuals that are otherwise socially isolated. High levels of formal participation among those with relatively many close social ties may not be pragmatically beneficial.

**Electronic supplementary material:**

The online version of this article (10.1007/s00127-020-01961-2) contains supplementary material, which is available to authorized users.

## Introduction

Poor mental health is a leading contributor to the burden of disease in Europe as well as around the world, with depression now being the single leading cause of global disability [1]. Widespread agreement exists that mental health status strongly influences physical health, and that mental health should be a key consideration in changing the general health status of a community or population [2]. The total cost of poor mental health in Europe has been estimated to be more than 4% of its GDP—or over €600 billion—across the EU [3]. Thus, it is imperative to identify protective factors that may prevent or delay poor mental health in late life to secure sustainability in European health and financial systems [4].

The concept of mental health is viewed as not just the absence of mental disorders (e.g., depression), but also the presence of positive mental states (e.g., quality of life—QoL) [5]. QoL has been defined as “the satisfaction of an individual’s values, goals, and needs through the actualization of their abilities or lifestyle” [6]. Mental health plays a major role in health behaviors, for example diet, sleep, and exercise, as well as risk behaviors, such as consumption of tobacco, alcohol and drugs, unsafe sexual behavior, or violent behavior [5], all of which may impact on overall health and the risk of developing chronic conditions. Depression may, for example, reduce or inhibit motivation to engage in healthy behaviors and to prioritize one’s own health [7]. Conversely, high QoL may promote good health by providing people with a sense of optimism and energy to engage in healthy behaviors [8].

A great deal of research has documented the fundamental role of social connectedness as a protective factor for mental health [9]. While social connectedness is a broad concept covering multiple types of being related to and engaged with the social environment, some research has focused specifically on *social participation*. Although agreement is lacking regarding specific definitions [10], informal social participation generally comprises an individual’s unstructured interactions with friends, family members, relatives, neighbors, coworkers, etc., while formal social participation refers to structured activities with others within established organizations, such as volunteering organizations, educational institutions, clubs, religious organizations, or political/civic groups [11, 12]. Numerous studies have demonstrated that formal social participation predicts well-being [13–15], while it also protects against the development of or increase in depression symptoms [16, 17]. Formal social participation is particularly interesting from a health promotion perspective, as it may be considered a behavioral gateway to other social constructs that have been shown to constitute protective factors for health. Formal social participation may, for example, be instrumental as a means of establishing lasting social relationships (e.g., conducive to social support) [18–21], increasing social capital (e.g., resources and benefits received through connections with others) [22], as well as fostering social identification (e.g., a sense of belonging) [20, 23].

If a general outcome of formal social participation is enhanced social connectedness, this may also imply that formal social participation would be particularly pertinent for individuals who are otherwise socially isolated. A number of studies have suggested that the benefit of formal social participation depends on contemporaneous social network characteristics. For example, studies utilizing data on American older adults showed moderating effects by social integration, where the mental health benefits of formal social participation were greatest for those who were less socially integrated [24, 25]. In a similar study involving American older adults, an association between informal (rather than formal) social participation and depression symptoms was investigated [26]. The authors found an inverse relationship between informal social participation and depression symptoms, but this association was much weaker among individuals with high quality social ties to spouses and children. These studies suggest that it may be particularly relevant to take social network size into account when estimating the potential benefits of social participation to mental health.

Therefore, the aim of this study was to investigate the extent to which social network size, i.e., the number of individuals’ close social ties moderates the relationship between formal social participation and mental health outcomes. We conducted a prospective study using data from two consecutive waves (2011, 2013) of the Survey of Health, Ageing, and Retirement in Europe (SHARE), a community-based survey of thirteen European countries. Our key variables were formal social participation, social network size (the number of close social ties), and validated measures for symptoms of depression and quality of life (QoL). To our knowledge, this is the first study to construct and assess a longitudinal moderation model with the variables of interest on a large multicountry dataset. It is essential to conduct such large-scale epidemiological studies to understand how—and especially for whom—formal social participation might serve as a protective factor against poor mental health in the general population. Based on the previous literature, we hypothesized that formal social participation would predict mental health, and that the number of close social ties would moderate identified associations. In particular, we hypothesized that formal social participation would—on average—positively predict QoL over time (Hypothesis 1), and negatively predict depression symptoms over time (Hypothesis 2). Further, we hypothesized that the number of close social ties would moderate the association between formal social participation and QoL (Hypothesis 3) as well as the association between formal social participation and depression symptoms (Hypothesis 4), showing that those individuals reporting relatively fewer close social ties would appear to benefit the most from formal social participation.

## Methods

**Fig. 1:**
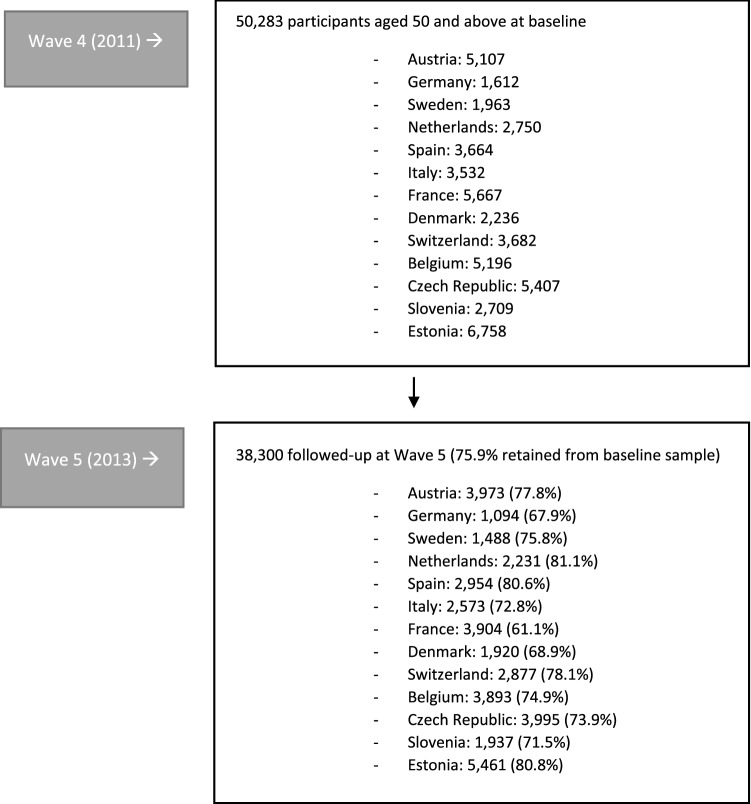
Flowchart of the study sample

The data came from wave 4 (2011) and 5 (2013) of the Survey of Health, Ageing and Retirement in Europe (SHARE) dataset (see Online Appendix 1 for more information). For the longitudinal analysis reported in this paper, we examined the 38,300 participants who took part in wave 4 and were followed-up in wave 5 (Fig. 1 shows the selection of the study sample). We chose to analyze data from waves 4–5 because wave 4 specifically assessed social networks (which was not included in subsequent waves), and because we wanted to conduct a longitudinal moderation analysis. Throughout the methods and results section, for ease of reference, waves 4 and 5 will be referred to as time 1 (T1) and time 2 (T2), respectively.

### Outcome 1: quality of life (QoL)

The Control, Autonomy, Self-realization, and Pleasure (CASP) scale examines significant aspects of QoL in older adulthood [27]. The SHARE questionnaire employs the CASP-12, a validated 12-item version of the scale (with a score range between 12 and 48) with higher scores indicating better QoL [28–30]. QoL was measured and operationalized (as a continuous measure) in the same way for both T1 and T2.

### Outcome 2: depression symptoms

Depression symptoms were measured using the EURO-D scale [31]. The EURO-D is a validated screening tool [32], that covers 12 symptom domains central to depression, such as lack of interest in things, irritability, sleep problems, lack of appetite and enjoyment, fatigue. Each domain is scored 1 if present, and 0 if absent. The total score is the sum of all the items, leading to a minimum score of 0 and a maximum score of 12, with higher scores indicating more symptoms of depression. Depression symptoms were measured and operationalized (as a continuous measure) in the same way for both T1 and T2.

### Predictor variable: formal social participation

Formal social participation was assessed by asking participants which of the following five activities they had engaged in the past 12 months: (1) done voluntary or charity work; (2) attended an educational or training course; (3) gone to a sport, social, or other kind of club; (4) taken part in activities of a religious organization (church, synagogue, mosque, etc.); or (5) taken part in a political or community-related organization. We used a scale (ranged 0–4) where a higher score indicated a higher frequency of formal social participation in at least one of the five types of social activities (for more information, see Online Appendix 1) [33].

### Potential moderating variable: social network size 

In order to identify the social network size (the number of close social ties), the respondents were asked to mention the name of persons with whom they discuss important personal matters. The total number of close relations in the social network is its *size*. It is possible to mention up to seven persons, however the boundary of maximum seven persons is only mentioned if it is reached. Less than 3% of the respondents reached this boundary. Most respondents stated one, two or three close social ties (28%, 25%, and 20% of the respondents, respectively). The variable was used as a continuous measure.

### Potential confounding variables

Potential confounders included age at baseline (T1, 2011), gender, marital status, education, individual household income, occupational status, country, activity limitations, and chronic conditions (described in Online Appendix 1).

### Statistical analysis

Stata v.13.1 was used to perform all analyses. A descriptive statistics analysis was conducted to illustrate the characteristics of the study sample. Multivariable linear regressions were conducted for each outcome, i.e., for QoL and depression symptoms respectively. In all models, formal social participation (continuous), social network size (continuous), and the interaction term (social participation × social network size) were entered into the models as the key predictor variables. Three models were conducted for each outcome: 1) one unadjusted model, 2) one unadjusted model that also included the interaction term, and 3) one adjusted model. Six variables, namely gender, marital status, education, occupation, activity limitations, and country, were entered into the models as categorical, and the remaining covariates were entered as continuous. To residualize the outcome variables and capture prediction of change, QoL at T1 was added to the covariates in the model predicting QoL at T2, and similarly, depression symptoms at T1 was added to the covariates in the model predicting depression symptoms at T2. Simple slopes for each social network size category (0, 1, 2, 3, 4, 5, 6, 7+) were calculated with the post hoc margins command in Stata. Conventionally, *p* values < 0.05 were considered statistically significant. For information on weighting and missing data, see Online Appendix 1

## Results

Table 1 shows the characteristics of the study sample at T1, along with descriptive statistics of QoL and depression symptoms at T1 and T2. The average age of the analytical sample was 66.2 (SD 9.7) years, and 56.8% were females. The average social network size was 2.5 (≈ 3 close social ties), which is in line with what we would expect based on previous research [34].

**Table 1:** Characteristics of the study sample

Characteristic	Category	*N* (%)
Time 1		
Unweighted *N*		38,300
Age (mean ± SD)		66.2 (9.7)
Gender	Female	21,746 (56.8)
Marital status	Married, living with spouse	24,866 (67.4)
	Registered partnership	557 (1.5)
	Married, separated from spouse	463 (1.3)
	Never married	2138 (5.8)
	Divorced	3499 (9.5)
	Widowed	5383 (14.6)
Education	None	1085 (2.9)
	Primary level	6664 (17.4)
	Lower secondary	7333 (19.5)
	Upper secondary	12,636 (33.6)
	Post-secondary nontertiary	1822 (4.9)
	First stage of tertiary	7693 (20.5)
	Second stage of tertiary	332 (0.9)
Individual household income	Lowest tertile	9484 (24.8)
	Middle tertile	11,235 (29.3)
	Highest tertile	10,944 (28.6)
	Missing	6673 (17.3)
Occupational status	Retired	21,507 (56.6)
	Employed or self-employed	10,428 (27.4)
	Unemployed	1133 (3.0)
	Permanent sick or disabled	1299 (3.4)
	Homemaker	3268 (8.6)
	Other	377 (0.9)
Activity limitations	Limited	18,178 (47.5)
Chronic conditions (mean ± SD)		1.5 (1.4)
Formal social participation	No formal social participation	19,172 (50.6)
	Active less than monthly in at least one activity	2664 (7.0)
	Active monthly in at least one activity	3593 (9.5)
	Active weekly in at least one activity	10,052 (26.5)
	Active daily in at least one activity	2422 (6.4)
Social network size (mean ± SD)		2.5 (1.6)
Depression symptoms^a^ (mean ± SD)		2.4 (2.2)
Quality of life^b^ (mean ± SD)		37.6 (6.3)
Time 2		
Depression symptoms^a^ (mean ± SD)		2.4 (2.2)
Quality of life^b^ (mean ± SD)		37.8 (6.3)

Data are unweighted *n* (%) unless otherwise specified

^a^Based on the 10-item EURO-D scale, range 0–12

^b^Based on the 12-item Control, Autonomy, Self-realization, Pleasure scale (CASP-12), range 12–48

### Quality of life

Table 2 shows the regression models predicting QoL at 2-year follow-up. According to these results, formal social participation and social network size both positively predicted QoL at 2-year follow-up, which were consistent with predictions, while the interaction term (formal social participation x social network size) also predicted QoL, but in the negative direction. Table 3 shows the graph depicting social network size moderating the association between formal social participation and QoL at 2-year follow-up, and reports the simple slopes by social network size. For individuals with a social network size of three or less, formal social participation positively predicted QoL. However, for individuals with a social network size of four and above, the associations between formal social participation and QoL were non-significant. Thus, formal social participation functioned as a positive predictor of QoL only for those individuals lacking a sizeable social network.

**Table 2:** The association between social participation, social network size and quality of life/depression symptoms at 2-year follow-up among older adults in Europe estimated by multivariable linear regression

	Quality of life		
	Unadjusted^a^		
	Coefficient	95% CI	*p* value
Social participation	1.01	0.96, 1.05	< 0.001
Social network size	0.31	0.27, 0.35	< 0.001

	Unadjusted incl. interaction term^b^		
	Coefficient	95% CI	*p* value
Social participation	1.17	1.09, 1.25	< 0.001
Social network size	0.41	0.35, 0.46	< 0.001
Interaction term	− 0.06	− 0.09, − 0.04	<0.001

	Adjusted^c^		
	Coefficient	95% CI	*p* value
Social participation	0.27	0.12, 0.43	0.001
Social network size	0.16	0.05, 0.26	0.005
Interaction term	− 0.05	− 0.09, − 0.0009	0.046

	Depression symptoms		
	Unadjusted^d^		
	Coefficient	95% CI	*p* value
Social participation	− 0.21	− 0.23, − 0.20	< 0.001
Social network size	− 0.03	− 0.04, − 0.01	0.001

	Unadjusted incl. interaction term^e^		
	Coefficient	95% CI	*p* value
Social participation	− 0.25	− 0.28, − 0.22	< 0.001
Social network size	− 0.05	− 0.07, − 0.03	< 0.001
Interaction term	0.01	0.005, 0.02	0.003

	Adjusted^f^		
	Coefficient	95% CI	*p* value
Social participation	− 0.08	− 0.14, − 0.03	0.003
Social network size	− 0.05	− 0.08, −0.006	0.024
Interaction term	0.02	0.007, 0.04	0.006

^a^Adjusted *R*^2^ = 0.07

^b^Adjusted *R*^2^ = 0.07

^c^The model (adjusted *R*^2^ = 0.48) adjusted for age, gender, marital status, education, income, occupational status, activity limitations, chronic conditions, and the quality of life at T1. Quality of life was based on the 12-item Control, Autonomy, Self-realization, Pleasure scale (CASP-12), range 12–48

^d^Adjusted *R*^2^ = 0.02

^e^Adjusted *R*^2^ = 0.02

^f^The model (adjusted *R*^2^ = 0.33) adjusted for age, gender, marital status, education, income, occupational status, activity limitations, chronic conditions, and the depression symptoms at T1. Depression symptoms were based on the 10-item EURO-D scale, range 0–12

**Table 3:**
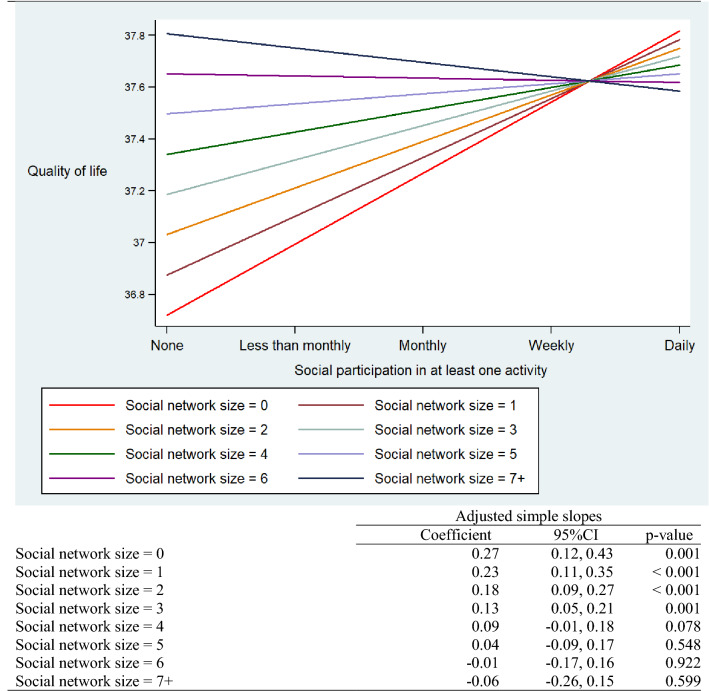
Adjusted simple slopes for social participation predicting quality of life at 2-year follow-up among older adults in Europe

Social network size = the number of close social ties. The model adjusted for age, gender, marital status, education, income, occupational status, activity limitations, chronic conditions, and quality of life at T1. Based on the 12-item Control, Autonomy, Self-realization, Pleasure scale (CASP-12), range 12–48. The *y*-axis represents predicted values for quality of life

### Depression symptoms

Table 2 shows the regression models predicting depression symptoms at 2-year follow-up. In this case, and as expected, formal social participation and social network size both inversely predicted depression symptoms at 2-year follow-up, while the interaction term also predicted depression symptoms, but in the positive direction. Table 4 shows the graph depicting social network size moderating the association between formal social participation and depression symptoms at 2-year follow-up, and reports the simple slopes by social network size. For individuals with a social network size of two or less, formal social participation negatively predicted depression symptoms. A nonsignificant association was noted for intermediate-sized social networks (three to six members), and interestingly, for individuals with a social network size of seven or more, formal social participation significantly and positively predicted depression symptoms. Combining the two results, we note that formal social participation was a significant beneficial predictor of changes in outcomes when individuals reported relatively small social networks. Additionally, the positive slope for individuals reporting seven or more members suggests that depression symptoms become elevated among individuals that are both embedded in a large social network and also engage in frequent formal social participation.

**Table 4:**
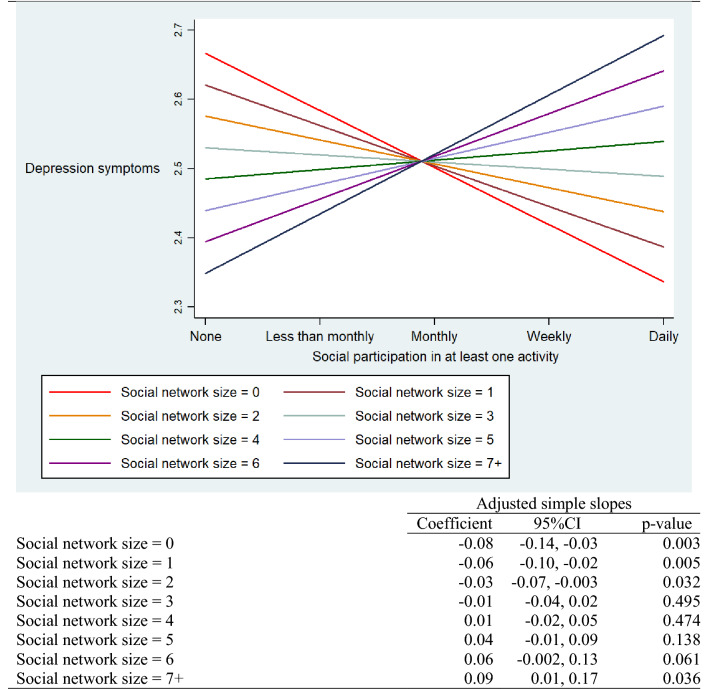
Adjusted simple slopes for social participation predicting depression symptoms at 2-year follow-up among older adults in Europe

Social network size = the number of close social ties. The model adjusted for age, gender, marital status, education, income, occupational status, activity limitations, chronic conditions, and quality of life at T1. Based on the 10-item EURO-D scale, range 0–12. The *y*-axis represents predicted values for depression symptoms

### Additional analysis

In order to make an analysis of the clinical significance of the results in terms of population prevalence estimates, we conducted similar models as those reported, but with logistic regressions including binary outcomes for (1) high QoL and (2) depression (see Online Appendix 1). The results of the logistic regression models were very similar to the linear regressions (Table A1). In terms of high QoL (Table A2), simple slopes for those with 0–4 close social ties were positively associated with high quality of life. Among individuals with 0–4 close social ties, the lowest level of social participation (none) at baseline had an estimated 41–47% population prevalence of high QoL at follow-up, while the highest level of social participation (daily) at baseline had an estimated 52–53% population prevalence of high QoL at follow-up. In terms of depression (Table A3), simple slopes for those with 0–2 close social ties were negatively associated with depression, while the simple slope for those with 7+ close social ties was positively associated with depression. Among individuals with 0–2 close social ties, the lowest level of social participation (none) at baseline had an estimated 30–32% population prevalence of depression at follow-up, while the highest level of social participation (daily) had an estimated 24–26% population prevalence of depression at follow-up.

## Discussion

With this study, we set out to investigate the moderating role of social network size, i.e., the number of close social ties in the association between formal social participation and mental health at 2-year follow-up among European community-dwelling older adults aged 50+ at baseline. Our results showed that formal social participation (i.e., active participation within volunteer organizations, educational institutions, clubs, religious organizations, or political/civic groups) on average predicted increases in QoL and reductions in depression symptoms. These findings supported our first two hypotheses, but it is crucial to note that they must be interpreted in the light of our post-hoc analyses. This leads us to our last two hypotheses, which were also supported. That is, only individuals reporting having relatively small social networks appeared to benefit from formal social participation. Among such individuals, the smaller the network, the greater the protective benefit appeared to be. Additionally in terms of depression symptoms, an unexpected finding was that among individuals reporting relatively numerous (seven or more) close social ties, formal social participation predicted an *increase* in depression symptoms. This counter-intuitive result likely reflects the fact that there is a natural limit to social network size in humans  [35, 36], implying that when the need for social connectedness has been met and the limit has been reached, individuals may not derive additional mental health benefits from engaging in formal social participation activities. On the contrary, it may be that too much social activity becomes a stress factor, leading people to being overwhelmed or experiencing feelings of guilt when social relationships are not tended to due to limited time available for each social tie.

### Strengths and limitations

The strengths of this study include the large sample size, the prospective design, the use of multicountry European nationally representative data, and validated scales for QoL and symptoms of depression. Some limitations should be considered before discussing the findings and their implications. First, these findings were based on self-reported data, which implies the possibility for self-report bias and issues pertaining to common-methods variance. Third, those participating in the baseline survey who did not participate in the follow-up survey were more likely to be older, unemployed, and have disability and lower education, as they were also more likely to report more chronic conditions, more depression symptoms, lower levels of social participation and smaller network sizes. Although we used weights to account for nonresponse and attrition throughout the waves, we cannot rule out some degree of bias introduced by missing data and loss to follow-up. Finally, it may be noted that the *R*^2^ estimates suggest that most of the variance in our models are explained by factors other than social participation and social network size. This may be expected given that mental health outcomes are affected by a myriad of factors (e.g., environment, personality, etc.). Although the predictor variables were significantly associated with the outcomes, the models indicate that the size of the effect may not be substantial, and this should be taken into account when interpreting the results. Owing to the large sample size, it is possible to detect small effects, which is a characteristic of many social processes.

### Contextualization of findings

Our results regarding QoL and depression symptoms were consistent with expectations, in that formal social participation was beneficial specifically among those with few close social ties, but did not appear to be beneficial among those with many close social ties. These results build on findings from the USA [24–26], demonstrating that the potential benefits of formal social participation among socially isolated older adults also applies across Europe. Our moderation results regarding depression symptoms also yielded an intriguing finding, namely that those individuals reporting many close social ties showed a positive association between formal social participation and depression symptoms. We did not expect that formal social participation would predict increases in depression symptoms for individuals with relatively numerous close social ties, but in retrospect this finding is perhaps not surprising given that previous research has suggested that there is a natural limit to social network size in humans [37, 38]. This limit would seem to be a consequence of natural constraints on available social time and resources [35], that is, committing to and maintaining quality social ties involves a significant amount of cognitive and emotional investment, which amounts to large quantities of time [39–41]. It may be that for individuals with many close social ties, engaging in formal social participation activities within established organizations may result in social over-commitment, emotional, and cognitive exhaustion, and fatigue, ultimately leading to compromised mental health.

In predicting depression symptoms, coefficients appeared to mirror each other below three and above four close social ties. This finding suggests that increasing levels of formal social participation may be just as detrimental in terms of depression symptom severity for those individuals reporting relatively many close social ties as it is beneficial for those with relatively few close social ties. The same mirroring pattern was not observed for QoL over the range of close social ties assessed here. However, a similar trend could be observed for QoL, and would perhaps have been evident if the upper limit for network size had been higher than seven. In that case, the pattern might be the same for symptoms of depression and QoL, only that the increase in depression symptoms would appear already with fewer social ties than the decrease in QoL. We can only speculate at this point, but it might be that the pressure of high levels of social activity more readily results in depression symptoms (e.g., restless sleep, lack of concentration), than it results in the decline in positive mental health (e.g., declines in optimism, meaningfulness). More research is warranted to explore this.

### Implications for policy and practice

Our findings have important implications for policy and practice in that efforts to foster social participation in communities may—if designed strategically—be worthwhile as a means to prevent the decline in mental health and well-being among the 50+ in Europe. Importantly, our findings indicate that interventions, initiatives or campaigns may potentially be made more effective if they particularly target people that are otherwise socially isolated. If our results are confirmed through intervention research, it is possible that such efforts could have significant implications for health and social systems by encouraging socially isolated individuals to engage in formal social participation. For this group specifically (those having relatively few social ties), our estimates suggest a 5–12% increase in the population prevalence of high QoL and a 4–8% reduction in the population prevalence of depression, if individuals were to move from low to high social participation. Given that those with relatively few close social ties (0–4 close social ties) account for over 70% of the 50+ population, such differences in prevalence rates—if achieved—would have major impact on a European scale. However, doing so would require broad, intersectoral collaborative efforts and partnerships between governmental and nongovernmental organizations [42–44].

Apart from the associated risk of declining mental health, socially isolated individuals are also at increased risk of many other critical outcomes, such as premature death [45] and dementia [46], and it is therefore imperative to intervene among these at-risk groups. Promoting formal social participation among such groups may be a promising approach for strategies to ameliorate these problems more efficiently. Efforts to assess social network size may make use of the instruments described in this study; alternatively, more advanced tools designed for practitioners to screen for restricted social networks already exist and have documented predictive validity in terms of various mental and physical health outcomes [47, 48]. Interventions to effectively alleviate late-life social isolation have been examined through meta-analytic reviews, with evidence supporting the effectiveness of educational and social activity group interventions that target specific groups, while the effectiveness of home visiting and befriending schemes has remained unclear [49]. Home visiting and befriending schemes are often popular within community efforts, but perhaps the best method to get people to connect with one another is to first get people involved in social activities they themselves consider meaningful. Doing so may subsequently promote social connectedness and a sense of belonging as a result of participating in these social activities [43]. For example, being part of singing groups or choirs has been shown to work especially well in terms of fostering connectedness because singing produces an immediate sense of social bonding through the release of endorphins [50], which in turn has been shown to benefit both physical and mental health and well-being [51]. Strategies may further be informed by the results of this study suggesting that it is important to maintain interactions with one’s close social ties or engage in formal social participation, but *not* necessarily both, and that there may be such a thing as ‘too much’ social activity, which may be detrimental to mental health.

## Conclusions

Our study examined the association between formal social participation (i.e., active participation within volunteer organizations, educational institutions, clubs, religious organizations, or political/civic groups) and mental health among older adults in Europe, and specifically the moderating influence of social network size (i.e., the number of close social ties) on this association. The moderation analyses showed that formal social participation among those with relatively few close social ties was positively associated with QoL and negatively associated with depression symptoms, but did not appear to be of benefit to those with relatively many close social ties. Contrary to our expectations, we found that formal social participation among individuals reporting seven or more close social ties was positively associated with depression symptoms. Strategies to foster social participation in communities may be worthwhile as a means to prevent the decline in mental health and well-being among the 50+ in Europe, and such strategies may in particular focus on segments of the population that are otherwise socially isolated. Campaigns and interventions may further take into account that formal and informal participation promote mental health, but high levels of both may not be pragmatically beneficial.

## Electronic supplementary material

Below is the link to the electronic supplementary material.

Supplementary material 1 (DOCX 80 kb)

## Data Availability

We do not have permission to share data.
