# Supplementary material for: The moderating role of social network size in the temporal association between formal social participation and mental health: a longitudinal analysis using two consecutive waves of the Survey of Health, Ageing and Retirement in Europe (SHARE)
Source: Soc Psychiatry Psychiatr Epidemiol. 2020 Oct 9;56(3):417–28. doi: 10.1007/s00127-020-01961-2 (PMC7904560; doi:10.1007/s00127-020-01961-2)
Supplement: Supplementary file 1 — Supplementary material 1 (DOCX 80 kb) [file 127_2020_1961_MOESM1_ESM.docx]

Appendix 1

*Sample*

SHARE is a European bi-annual, cross-national and longitudinal research project collecting nationally-representative data among community-dwelling participants aged 50 years and older at this study’s baseline (i.e. wave 4, 2011). To ensure that all waves are representative of the respective countries, the SHARE survey includes refreshment samples to increase net sample size and compensate for attrition in the longitudinal samples. Waves 4 and 5 were conducted in 13 out of 20 countries participating in SHARE: Austria, Germany, Sweden, the Netherlands, Spain, Italy, France, Denmark, Switzerland, Belgium, Czech Republic, Slovenia, and Estonia. Wave 4 response rates varied from 39.5% for the Netherlands to 63% for Hungary (1). Ethics approval for the SHARE project was granted by the Ethics Council of the Max-Planck-Society for the Advancement of Science.

*Formal social participation*

Formal social participation was assessed by asking participants which of the following five activities they had enacted in the past 12 months: 1) Done voluntary or charity work; 2) Attended an educational or training course; 3) Gone to a sport, social, or other kind of club; 4) Taken part in activities of a religious organization (church, synagogue, mosque, etc.); or 5) Taken part in a political or community-related organization. Participants were first asked to indicate whether or not they had participated in any of the five activities (0 = no; 1 = yes), and then were subsequently asked about the frequency of the mentioned activities (almost daily; almost every week; almost every month; less often). A scale (2) for frequency of formal social participation was created by combining both items where 4 = active almost daily in at least one activity; 3 = active almost every week in at least one activity; 2 = active almost every month in at least one activity; 1 = active less than monthly at least in one activity; 0 = no formal social participation.

*Potential confounding variables*

Several sociodemographic variables were included as potential confounding variables. Demographics included age at baseline (T1, 2011) and gender (male, female). Marital status had the following six categories: Married and living together with spouse; registered partnership; married and living separated from spouse; never married; divorced; or widowed. Education was assessed according to the International Standard Classification of Educational Degrees (ISCED-97) with the following seven categories: None; primary level of education; lower secondary level of education; upper secondary level of education; post-secondary non-tertiary; first stage of tertiary education; or second stage of tertiary education. Individual household income was categorized into tertiles: low; middle; high. Since 17.3% of the data was missing for income, a ‘missing’ category was created in order to not lose a substantial amount of data in the analyses due to this covariate. Occupational status was categorized as: retired; employed or self-employed; unemployed; permanently sick or disabled; homemaker; or other. In terms of national setting, a categorical variable included all individual countries.

Other covariates included the Global Activity Limitation Indicator (GALI), which is a single-item survey instrument to assess health-related activity limitations (3). The data for GALI were dichotomized into limited and not limited. Number of chronic conditions was assessed by the question “has a medical doctor ever told you that you have [condition]?” Responses included 13 conditions such as cardiovascular illnesses, cancer, arthritis, neurological disorders (2).Chronic conditions were coded dichotomously, 0 = no; 1 = yes, and the number of chronic conditions were subsequently summed (range 0 – 13), with higher scores representing more chronic conditions.

*Weighting*

In all analyses, the sample weighting was taken into account to obtain nationally representative estimates using the SHARE-provided longitudinal weight. The weight was calibrated against the total national population by gender and age-groups for each country in the sample, and further calibrated to compensate for both problems of unit non-response in the baseline sample, as well as problems of attrition in the samples of the subsequent waves (4).

*Missing data*

All statistical models were based on the sample with no missing data (complete case analysis), and the proportion of missing data within the study sample (i.e. all individuals followed through T1-T2, N=38,300) were as follows: QoL 5.2% (T1) and 5.8% (T2); depression symptoms 2.3% (T1) and 3.0% (T2); social network size 0% (T1); social participation 1.0% (T1); gender 0% (T1); age 0% (T1); marital status 3.6% (T1); education 1.9% (T1); income 0% (T1); occupation 0.8% (T1); activity limitations 0.2% (T1); chronic conditions 0.2% (T1), country 0% (T1).

*Additional analysis – logistic regressions*

We conducted additional logistic regressions to show the clinical significance of reported associations. In accordance with previous SHARE publications, we used a cut-point for high QoL as a CASP-12 score of ≥39 (5), and a cut-point for depression as a EURO-D score of >3 (6). We used these cut-points since we were interested in opposite ends of a single mental health continuum. The N(%) for high QoL was 17,997 (49.6%) at W4, and 18,168 (50.4%) at W5. The N(%) for depression was 10,240 (27.3%) at W4, and 10,052 (27.1%) at W5.

| **Table A1.** The association between social participation, social network size and high quality of life or depression at 2-year follow-up among older adults in Europe estimated by multivariable logistic regression. | | | | | | |
| --- | --- | --- | --- | --- | --- | --- |
|  |  |  |  | **High Quality of Life** | | |
|  |  |  |  |  | Unadjusted^a^ |  |
|  |  |  |  | Coefficient | 95%CI | p-value |
| Social participation |  |  |  | 1.29 | 1.25, 1.33 | < 0.001 |
| Social network size |  |  |  | 1.10 | 1.07, 1.14 | < 0.001 |
|  |  |  |  | Unadjusted incl. interaction term^b^ | | |
|  |  |  |  | Coefficient | 95%CI | p-value |
| Social participation |  |  |  | 1.29 | 1.20, 1.39 | < 0.001 |
| Social network size |  |  |  | 1.11 | 1.06, 1.17 | < 0.001 |
| Interaction term |  |  |  | 0.97 | 0.95, 0.99 | 0.016 |
|  |  |  |  |  | Adjusted^c^ |  |
|  |  |  |  | Coefficient | 95%CI | p-value |
| Social participation |  |  |  | 1.19 | 1.10, 1.28 | 0.001 |
| Social network size |  |  |  | 1.09 | 1.03, 1.15 | 0.003 |
| Interaction term |  |  |  | 0.98 | 0.95, 0.998 | 0.038 |
|  |  |  |  | **Depression** | | |
|  |  |  |  | Unadjusted^d^ | | |
|  |  |  |  | Coefficient | 95%CI | p-value |
| Social participation |  |  |  | 0.86 | 0.83, 0.89 | < 0.001 |
| Social network size |  |  |  | 0.99 | 0.96, 1.03 | 0.625 |
|  |  |  |  | Unadjusted incl. interaction term^e^ | | |
|  |  |  |  | Coefficient | 95%CI | p-value |
| Social participation |  |  |  | 0.79 | 0.74, 0.85 | < 0.001 |
| Social network size |  |  |  | 0.95 | 0.91, 0.997 | 0.038 |
| Interaction term |  |  |  | 1.03 | 1.01, 1.06 | 0.005 |
|  |  |  |  | Adjusted^f^ | | |
|  |  |  |  | Coefficient | 95%CI | p-value |
| Social participation |  |  |  | 0.87 | 0.81, 0.95 | 0.001 |
| Social network size |  |  |  | 0.94 | 0.91, 0.99 | 0.015 |
| Interaction term |  |  |  | 1.04 | 1.01, 1.06 | 0.003 |
| ^a^ Adjusted R^2^ = 0.03.  ^b^ Adjusted R^2^ = 0.03.  ^c^ The model (adjusted R^2^ = 0.25) adjusted for age, gender, marital status, education, income, occupational status, activity limitations, chronic conditions, and the quality of life at T1. Quality of life was based on the 12-item Control, Autonomy, Self-realization, Pleasure scale (CASP-12).  ^d^ Adjusted R^2^ = 0.01.  ^e^ Adjusted R^2^ = 0.01.  ^f^ The model (Adjusted R^2^ = 0.19) adjusted for age, gender, marital status, education, income, occupational status, activity limitations, chronic conditions, and the depression symptoms at T1. Depression was based on the 10-item EURO-D scale. | | | | | | |

| **Table A2.** Adjusted^a^ simple slopes for social participation predicting population averages of high quality of life^b^ at 2-year follow-up among older adults in Europe. | | | |
| --- | --- | --- | --- |
| 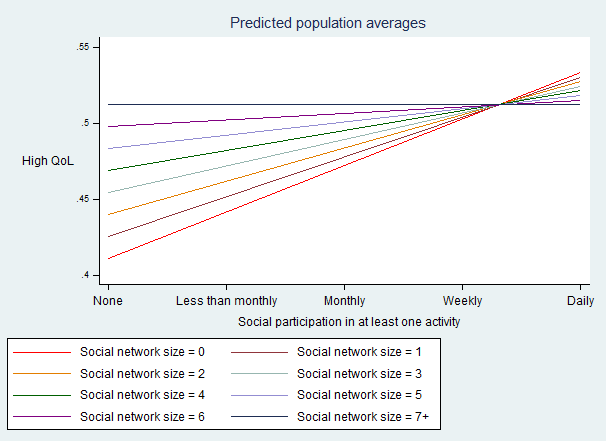 | | | |
|  | Adjusted simple slopes | | |
|  | Coefficient | 95%CI | p-value |
| Social network size = 0 | 0.030 | 0.017, 0.042 | < 0.001 |
| Social network size = 1 | 0.026 | 0.016, 0.035 | < 0.001 |
| Social network size = 2 | 0.021 | 0.014, 0.029 | < 0.001 |
| Social network size = 3 | 0.017 | 0.010, 0.024 | < 0.001 |
| Social network size = 4 | 0.013 | 0.004, 0.022 | 0.004 |
| Social network size = 5 | 0.009 | -0.003, 0.021 | 0.149 |
| Social network size = 6 | 0.004 | -0.011, 0.020 | 0.581 |
| Social network size = 7+ | -0.00006 | -0.019, 0.019 | 0.995 |
| The y-axis represents predicted population averages for high quality of life. Social network size = number of close social ties.  ^a^ The model adjusted for age, gender, marital status, education, income, occupational status, activity limitations, chronic conditions, and high quality of life at T1.  ^b^ Based on the 12-item Control, Autonomy, Self-realization, Pleasure scale (CASP-12). | | | |

| **Table A3.** Adjusted^a^ simple slopes for social participation predicting population averages of depression^b^ at 2-year follow-up among older adults in Europe. | | | |
| --- | --- | --- | --- |
| 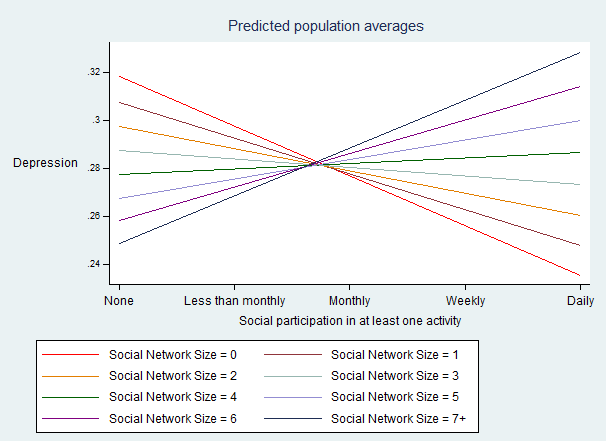 | | | |
|  | Adjusted simple slopes | | |
|  | Coefficient | 95%CI | p-value |
| Social network size = 0 | -0.021 | -0.034, -0.009 | 0.001 |
| Social network size = 1 | -0.015 | -0.025, -0.006 | 0.001 |
| Social network size = 2 | -0.009 | -0.016, -0.002 | 0.009 |
| Social network size = 3 | -0.004 | -0.010, 0.003 | 0.292 |
| Social network size = 4 | 0.002 | -0.006, 0.010 | 0.583 |
| Social network size = 5 | 0.008 | -0.003, 0.019 | 0.146 |
| Social network size = 6 | 0.014 | -0.0003, 0.028 | 0.055 |
| Social network size = 7+ | 0.020 | 0.002, 0.037 | 0.030 |
| The y-axis represents predicted population averages for depression. Social network size = number of close social ties.  ^a^ The model adjusted for age, gender, marital status, education, income, occupational status, activity limitations, chronic conditions, and depression at T1.  ^b^ Based on the 12-item Control, Autonomy, Self-realization, Pleasure scale (CASP-12). | | | |

References

1. Abduladze L, Balster E, Börsch-Supan A, Czaplicki C, Das M, De Luca G, et al. SHARE wave 4: innovations & methodology: Munich center for the economics of aging; 2013.

2. Santini ZI, Jose PE, Koyanagi A, Meilstrup C, Nielsen L, Madsen KR, et al. Formal social participation protects physical health through enhanced mental health: A longitudinal mediation analysis using three consecutive waves of the Survey of Health, Ageing and Retirement in Europe (SHARE). Social Science & Medicine. 2020;251:112906.

3. Van Oyen H, Bogaert P, Yokota RTC, Berger N. Measuring disability: a systematic review of the validity and reliability of the Global Activity Limitations Indicator (GALI). Archives of Public Health. 2018;76(1):25.

4. De Luca G, Rossetti C. Stata program to compute calibrated weights from scientic usefile and additional database. Deliverable 2.10 of the SERISS project funded under the European Union’s Horizon 2020 research and innovation programme GA No: 654221 2018. Available from: <https://seriss.eu/wp-content/uploads/2018/08/SERISS-Deliverable-2.10-Stata-program-for-calibrated-weights.pdf>.

5. von dem Knesebeck O, Hyde M, Higgs P, Kupfer A, Siegrist J. Quality of life and well-being. In: Börsch-Supan A, Brugiavini A, Jürges H, Mackenbach J, Siegrist J, Weber G, editors. Health, Ageing and Retirement in Europe - First Results from the Survey of Health, Ageing and Retirement in Europe. Mannheim: MEA; 2005. p. 199-203.

6. Dewey M, Prince M. Mental health. In: Börsch-Supan A, Brugiavini A, Jürges H, Mackenbach J, Siegrist J, Weber G, editors. Health, ageing and retirement in Europe - First results from the survey of health, ageing and retirement in Europe: Mannheim Research Institute for the Economics of Aging; 2005. p. 108-17.
